# Disease knowledge and attitudes during the COVID-19 epidemic among international migrants in China: a national cross-sectional study

**DOI:** 10.7150/ijbs.47075

**Published:** 2020-09-04

**Authors:** Cheng Wang, Qi Tian, Peizhen Zhao, Mingzhou Xiong, Carl A Latkin, Yiqun Gan, Brian J Hall, Bin Yang

**Affiliations:** 1 Dermatology Hospital, Southern Medical University, Guangzhou, Guangdong, China.; 2 Southern Medical University Institute for Global Health and Sexually Transmitted Diseases, Guangzhou, Guangdong, China.; 3 Guangzhou Health Information Center, Guangzhou, Guangdong, China.; 4 Department of Health, Behavior and Society, Johns Hopkins Bloomberg School of Public Health, Baltimore, MD, USA.; 5 School of Psychological and Cognitive Sciences, Peking University, Beijing, China.; 6 Global and Community Mental Health Research Group, New York University (Shanghai), Shanghai, China.

**Keywords:** knowledge, attitude, COVID-19, international migrants

## Abstract

**Background:** There are more than 258 million international migrants worldwide and the majority reside in countries with ongoing novel coronavirus disease 2019 (COVID-19) epidemic outbreaks. International migrants may not receive adequate and timely disease information during epidemics, increasing vulnerability to disease transmission. This is one of very limited studies focusing on international migrants' COVID-19 prevention knowledge and attitudes during the epidemic.

**Methods:** A national cross-sectional online survey was conducted across 100 cities and 26 regions in China from February 17 and March 1, 2020. The sample included 1,426 international migrants representing 77 countries and 6 continents. Knowledge was defined as the number of correct responses to questions about COVID-19. Attitudes included worries, expectations, and general preparedness. Multivariable ordinal logistic regressions evaluated correlates of knowledge and attitudes including information channels and preferences, and trust in Chinese institutions and groups.

**Results:** Just half of the sample, 730/1426 (51.2%) had a good level of knowledge and 656/1426 (46.0%) had a positive attitude towards the COVID-19 epidemic. Knowledge was associated with receiving information through social media (aOR: 2.0, 95%CI: 1.2-3.2), the Internet (aOR: 1.4, 95%CI: 1.2-1.8), the community (aOR: 1.5, 95%CI: 1.2-1.8), and encountering language barriers when receiving medical services (aOR: 0.8, 95%CI: 0.7-1.0). Positive attitude was associated with the level of trust in various Chinese institutions and groups.

**Conclusions:** Roughly half of the sample reported inadequate knowledge and poor attitudes toward prevention and control of COVID-19. Tailored public health campaigns are needed to ensure that international migrants possess adequate knowledge to protect their health during future epidemics and disasters.

## Introduction

On March 13, 2020, the World Health Organization (WHO) upgraded the status of the novel coronavirus disease 2019 (COVID-19) outbreak from an epidemic to pandemic 1. As of June 7, 2020, 6,799,713 confirmed cases, and 397,388 deaths were reported in 215 countries worldwide 2. China was the first and one of the worst-hit countries overall, with deaths surpassing 4,000 and confirmed cases reaching 84,629 2. After a country-wide effort to stop the epidemic in China, the number of confirmed cases reported each day has declined as of February 19, 2020. 3. China has begun to ease internal epidemic-control efforts towards international migrants and travelers since March 3, 2020 3, 4.

In 2017, the number of international migrants was estimated to be 258 million worldwide 5. Of whom, over 95% reside in countries with ongoing COVID-19 outbreaks 2. In China, there are an estimated 978,046 international migrants 5. Compared with the local population, international migrants encounter many barriers in accessing health services, including language and cultural differences, discrimination from local residents, precarious legal status, mental disorders and a lower quality of life 6-8. This situation could worsen during the COVID-19 epidemic. Addressing the health needs of international migrants has become an urgent public health priority during the rapid spread of COVID outbreak worldwide. Adequate knowledge and risk communication strategies about the coronavirus were key to protect the health of international migrants in China 7. However, there is no study on international migrants' knowledge as well as their attitudes associated with the COVID-19 epidemic.

Findings from the SARS outbreak in 2003 suggest that knowledge and attitudes towards infectious diseases and the trust in the local government institutions and groups, are associated with poorer adherence to epidemic control measures 9. The unprecedented nature of this epidemic provides an opportunity to inform future public health preparedness. The aim of this national study was to explore the level of knowledge and attitudes towards the COVID-19 epidemic and their determinants among international migrants in China.

## Methods

### Study Design and participants

An online cross-sectional survey was conducted using convenience sampling between February 17 and March 1, 2020. We partnered with community leaders from three active international migrant community-based organizations (CBO) in Guangzhou to help with the recruitment (representing the countries Nigeria, Ghana, and Zimbabwe). They mobilized their peer network and disseminated a link to the online survey to potential participants on Wechat (a popular Chinese messaging app). Participants entered the survey by clicking on the link, which directed them to a survey website hosted by WenJuanXing (Changsha Haoxing Information Technology Co., Ltd., China). Participants were then encouraged to recruit other eligible individuals to the study and informed they would receive 1.5 US dollars for each effective online referral. The survey questionnaire was available in English and created based on formative research, which included discussions with CBO stakeholders, policymakers, and experts on international migrants and survey pilot testing with 20 international migrants. We engaged these stakeholders and community members to ensure that the survey was clear and well understood by our target population. This pilot data was not included in the final analysis.

All participants who clicked the link for the survey were screened for eligibility. Inclusion criteria were being born in a country outside of China, aged 16 or over, cumulatively living in China for one month or more and staying in China between December 2019 and February 2020. The survey was restricted to one phone number and a single device to minimize the risk of people participating multiple times. Participants received 2 US dollars on completion of the study.

## Measures

### Knowledge about COVID-19

Knowledge of COVID-19 was measured by 7 items developed using the World Health Organization's COVID-19 advice for the public 10: the symptoms after contracting COVID-19, the signs indicating seeking health care immediately, the outcomes caused by COVID-19, transmission routes, prevention strategies, quarantine period, and availability of specific drug or vaccine. Each of the 7 knowledge items was coded 0 for an incorrect answer and 1 for a correct answer, and the total score ranged from 0 to 7. A higher score indicated better knowledge of COVID-19. We categorized individuals' knowledge into poor, moderate and good if they received scores of 0-3, 4-5, and 6-7, respectively. In the current study, the Cronbach's alpha for the scale was 0.76.

### Attitude towards the COVID-19 epidemic

Attitude towards the COVID-19 epidemic was measured by 5 items (strongly agree/agree/disagree/strongly disagree): being confident in knowing how to protect yourself from the COVID-19, worrying about contracting COVID-19, worrying about loved ones/friends contracting COVID-19, feeling helpless to prevent COVID-19, being confident the COVID-19 will end soon. Each item was coded 0 if they disagreed and 1 if participants agreed. The total score ranged from 0-5. A higher score indicated a more positive attitude toward COVID-19 prevention and control. We categorized individuals' attitudes into negative, neutral, and positive if they received scores of 0-2, 3, and 4-5, respectively. The Cronbach's alpha for the scale was 0.81.

### Information channels and preferences for COVID-19 information

Information channels and preferences for COVID-19 information included 5 items: channels of information received, preferred channels for receiving information, preferred types of information, barriers to receive medical services, and rating of the quality of medical service regarding COVID-19 in China.

### Trust toward Chinese institutions and groups during the COVID-19 outbreak

Trust toward Chinese institutions and groups included 7 items 11: the Central Government, the department that is responsible for health, the department that handles immigration, the hospital system, doctors and medical professionals, the information you are receiving about the COVID-19, the police, and the Chinese people. The scoring of each item of the scale ranged from 0 to 100 with intervals of 10. A score of 0 implies no trust at all, and 100 indicated complete trust. We categorized individuals' trust into “high trust,” “moderate trust,” and “low trust” if they received scores of 80-100, 60-80, and less than 60, respectively. This measure of trust in Chinese institutions and groups was deleted by the survey platform after responses were recorded from 868 individuals, because of political sensitivity.

### Experiences with COVID-19

Experiences with COVID-19 included 5 items: possibly being infected with COVID-19, receiving any medical services regarding COVID-19 in China, plan to seek medical screening for diagnosis, reasons for not testing or seeking diagnostic treatment, and plans to leave China because of COVID-19.

### Social-demographic variables

Socio-demographic information included: gender, age, marital status, education, income, religion, home country, and living arrangement in China, purpose of migration, health insurance, and health condition.

### Statistical analysis

Descriptive statistics describe the distribution of the sample regarding participant characteristics, knowledge, attitude, experiences, information channels and preferences, trust in Chinese institutions and groups. Chi-square test was performed to compare differences in knowledge of and attitudes towards COVID-19 across subgroups of respondents by sociodemographic characteristics.

Univariate and multivariable ordinal logistic regressions were conducted to explore the factors associated with knowledge and attitude to COVID-19 epidemic. The knowledge outcome was categorized into poor, moderate and good. The attitude outcome was categorized into negative, neutral and positive. In the multivariable ordinal models, we adjusted for gender, age, marital status, education, income, religion, original country, and reasons for migration. Statistical significance was defined as *p*<0.05. Analyses were performed using SAS 9.4 (SAS Institute Inc., Cary, NC).

## Results

**Figure 1:**
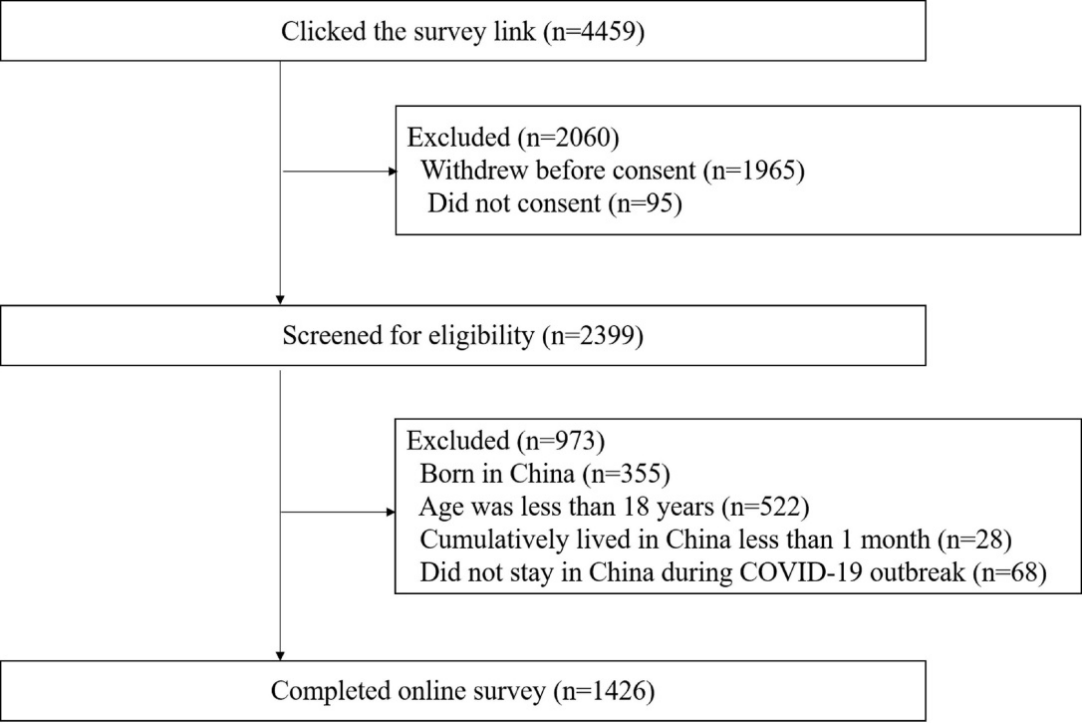
Flowchart diagram of study population.

Overall, 2494 people accessed the platform, and ninety-five were excluded for not signing the consent form. Among the remaining 2399 individuals, 973 did not meet eligibility requirements (355 were born in China, 522 were less than 18 years old, 28 cumulatively lived in China less than 1 month, and 68 did not stay in China during COVID-19 outbreak). A total of 1426 individuals completed the online survey. (**Figure 1**) These individuals were located in 100 cities of 26 provinces and regions of China and originated from 77 countries and 6 continents (see Supplementary participant's geographic distribution and origin countries). There were 868 individuals who completed the items on trust in Chinese institutions and groups before those items were deleted by the survey platform.

The majority of participants were male (60.9%), between 16 and 35 years old (89.6%), never married (86.7%), Christian (64.4%), had a college degree or higher (58.0%), had an annual income less than $2000 USD (62.7%), and originated from African countries (73.6%). More than half reported coming to China for study (61.2%), with a cumulative stay in China for one year or above (77.4%), and they stayed in China for 3 months between December 2019 and February 2020 (79.7%), during the epidemic period. Most individuals reported having health insurance in China (83.5%), not being diagnosed with any infectious disease in the past year (75.0%), and not being diagnosed with COVID-19 (99.1%) (**Table 1**).

### Knowledge to COVID-19

Correct answers for the 7 items on COVID-19 knowledge ranged from 51.1%-96.1%. Around half of the international migrants (51.2%, 730/1426) had a good level knowledge about COVID-19. Most individuals had a correct understanding of the symptoms of COVID-19 (74.0%, 1055/1426), the signs of indicating seeking health care immediately (85.3%, 1217/1426), the health sequela of COVID-19 (88.1%, 1256/1426), transmission routes (96.1%, 1370/1426), and quarantine period (94.0%, 1340/1426). Only around half of individuals reported a correct answer on the availability of specific drugs or vaccine for COVID-19 (53.3%, 760/1426) and the prevention strategies on COVID-19 (51.1%, 729/1426). In particular, reducing contact with wild animals (64.7%, 923/1426), and keeping rooms well ventilated (65.9%, 939/1426) were prevention strategies that most people answered incorrectly (**Table 2**).

Comparisons of knowledge by participant characteristics showed that eight variables were significantly different: gender, age, origin country, religion, reasons for migration, living arrangement in China, the number of days staying in China between December 2019 and February 2020, whether they had an infectious disease in the past year, and whether they were diagnosed with COVID-19 (*p*<0.001) (**Table 1**).

### Attitude towards the COVID-19 epidemic

A total of 46.0% (656/1426) of international migrants had a positive attitude overall in preventing and addressing COVID-19. Most individuals reported being confident in knowing how to protect themselves from COVID-19 (89.2%, 1272/1426), being confident that the COVID-19 epidemic will end soon (88.3%, 1259/1426), not worrying about themselves (58.4%, 833/1426) contracting COVID-19 and not feeling helpless to prevent COVID-19 (64.7%, 922/1426). However, more than half (57.9%, 825/1426) reported worrying about loved ones/friends contracting COVID-19 (**Table 2**).

Among the 126 (8.8%, 126/1426) individuals who reported that they might have been infected with COVID-19, only 34.9% (44/126) reported that they planned to seek medical screening for diagnosis. The most common reason for not testing or seeking diagnostic treatment was fear of the virus (43.7%, 55/126). Around two-thirds of the sample (67.5%, 962/1426) reported not planning to leave China because of COVID-19 (**Table 2**).

Comparisons of attitudes of different participant demographic characteristics showed that only two variables were significantly different: reasons for migration, and living arrangement in China (*p*<0.05) (**Table 1**).

### Trust in Chinese institutions and groups

Overall, more than half the participants reported a high level of trust toward Chinese institutions and groups (52.4%, 455/868). The doctors and medical professionals were the groups to which most individuals gave high trust (69.7%, 605/868), followed by the police (62.1%, 539/868). Individuals reported high trust (68.2%, 592/868) in the department that handles immigration, followed by the hospital system (66.6%, 578/868). Levels of trust in Chinese institutions and groups was associated with knowledge (*p*<0.001) and attitudes (*p*<0.001) (**Table 3**).

### COVID-19 information channels and preferences

The most common channel of receiving information was from Wechat (94.5%, 1348/1426), followed by friends (59.5%, 849/1426). The most preferred information to know was how to cure the disease (66.8%, 952/1426), followed by where the virus came from (65.0%, 927/1426). The most common barrier to receive medical services was language communication (55.1%, 786/1426). More than half (56.9%, 812/1426) rated the quality of medical service received regarding COVID-19 in China as good (**Table 4**).

### Factors correlated with knowledge about the COVID-19

In the multivariable ordinal logistic regression analyses adjusted for gender, age, legal marital status, highest educational attainment, annual income (USD), original country, religion, and reasons for migration, the odds of moving from a poor level of knowledge to a moderate or good level of knowledge among individuals who received information through social media were 2 times (aOR: 2.0, 95%CI: 1.2-3.2) greater than those not receiving information through social media. Two other factors were also positively associated with a higher odds of having a good level of knowledge: Internet (website/app related to news) (aOR: 1.4, 95%CI: 1.2-1.8), and community (community/friends/leaflet) (aOR: 1.5, 95% CI: 1.2-1.8). The other factor of individuals who encounter language barriers when receiving medical services was negatively associated with a higher likelihood of having a good level of knowledge (aOR: 0.8, 95% CI: 0.7-1.0) (**Table 5**).

### Factors correlated with Attitude towards the COVID-19 epidemic

In the multivariable ordinal logistic regression analyses adjusted for gender, age, legal marital status, highest educational attainment, annual income (USD), original country, religion, and reasons for migration, the odds of moving from negative attitude towards the COVID-19 epidemic to neutral or positive attitude in individuals rating the quality of medical service regarding COVID-19 in China as moderate were 1.5 times (aOR: 1.5, 95% CI: 1.1-2.0) greater than those who rated the quality as poor. Nine other factors were also positively associated with a higher odds of having a positive attitudes: rating the quality of medical service regarding COVID-19 in China as good (aOR: 1.7, 95% CI: 1.3-2.2), high level of trust in the Central Government (aOR: 1.6, 95% CI: 1.1-2.2), the department that handles health (aOR: 1.6, 95% CI: 1.1-2.3), the department that handles immigration (aOR: 1.5, 95% CI: 1.0-2.2), the hospital system (aOR: 1.8, 95% CI: 1.2-2.6), doctors and medical professionals (aOR: 2.1, 95% CI: 1.4-3.1), the information received about COVID-19 (aOR: 1.8, 95% CI: 1.3-2.5), the police (aOR: 1.7, 95% CI: 1.2-2.4), and the Chinese people (aOR: 1.7, 95% CI: 1.3-2.3) (**Table 5**).

## Discussion

International migrants' health needs and their access to health care should be a priority during the COVID-19 epidemic 7. This is one of very limited studies evaluating the overall knowledge and attitudes about COVID-19 among diverse migrants living in China during this public health emergency. Our data suggests that greater efforts are needed to improve the knowledge and attitudes of international migrants about COVID-19. Findings from this study provides insights on how quality health care can be improved, and the critical need to expand public health messaging and risk communication in China that includes international migrants.

We found that many international migrants in China did not have a good level of knowledge about the COVID-19, even in the middle of the epidemic. The correct answer rates of COVID-19 knowledge in this study were much lower than previously reported among Chinese residents 12. This low rate suggests that the Chinese government placed greater emphasis on health promotion and risk communication among Chinese residents at this stage of the epidemic, and a relative lack of tailored public health campaigns (e.g, materials in multiple languages) for international migrants. Our study showed that social media was the main channel of receiving information among international migrants in China regarding COVID-19, which could be a useful platform for providing timely information and other forms of social support during the epidemic. However, in the absence of reliable information in their own language, social media can also easily spread inaccurate information which may lead to panic and delayed visits to health centers among international migrants 13. Therefore, assessing the accuracy of information and providing health information in multiple languages tailored for the international migrants are needed for a more inclusive future public health response.

Our study showed that less than half of international migrants in China held a positive attitude about prevention and control of COVID-19 overall. Although most individuals were confident that COVID-19 would finally be successfully controlled, which is consistent with a previous study in the Chinese residents,^12^. many international migrants were still worrying about loved ones/friends contracting COVID-19. We found that only a third of individuals who reported the possibility of having been infected with COVID-19 planned to seek medical screening for diagnosis. The most common reason for not seeking medical screening was fear of the virus (43.7%), which highlights the importance of providing tailored support to this population. Measures to enhance inclusion of migrants in emergency planning may include providing daily updates about the COVID-19 epidemic in multiple languages, enhancing support systems, eliminating stigma associated with the epidemic, and providing psychosocial services (e.g., telephone-based and internet-based counseling) 14. These measures may also help to reduce the incidence of mental health disorders caused by the stress of the COVID-19 epidemic 14.

Our study found that most international migrants showed a high level of trust in Chinese institutions and groups during the COVID-19 epidemic overall. This may be related to the Chinese governments' unprecedented COVID-19 control measures such as traffic limits throughout China, shutdowns of cities and counties of Hubei Province, and the concerted efforts from across the country. Results from this study showed that individuals with a higher level of trust in Chinese institutions and groups were more likely to have a positive attitude toward COIVD-19. However, our study also showed that many international migrants were dubious about received information regarding COVID-19 and Chinese people. This may be due to cultural differences, a language barrier, or stigma and discrimination 15. More public health communications are needed to fill these gaps, as previous studies showed that some migrants in China rely on their own community, rather than government sponsored public health programming 16.

Our study has several limitations. First, participants were recruited exclusively online, likely resulting in selection bias. However, online technology has rapidly increased in use to recruit hard-to-reach populations 17 because they can efficiently recruit large samples 18 and have some automation processes which facilitate study implementation 19. A previous study showed that results from an online survey on men who have sex with men (MSM) could be quantitatively generalized to a national, cross-sectional survey dataset on MSM in China 20. Second, we were only able to record 868 individuals' responses on the trust in Chinese institutions and groups in this study. Although the respondents were similar to those who were unable to answer these questions, this may have introduced bias in our estimates. The survey questionnaire was only available in English, which might have led to selection bias. Third, all the data were collected through self-report, which may be prone to information bias. Forth, a majority of participants were from African countries. The results of this study may not be generalizable to migrants from different countries of origin.

## Conclusions

Many international migrants in China did not have good knowledge and positive attitudes toward prevention and control during the COVID-19 epidemic in China. Public health outreach to this community should be improved and tailored public health campaigns are needed to ensure that international migrants possess adequate knowledge to protect their health during future epidemics and disasters. International migrants showed a high level of trust in Chinese institutions and groups during the rapid spread of the COVID-19 epidemic outbreak and trust was a key determinant affecting international migrants' attitudes to COVID-19. The global population of migrants will continue to rise, and host countries should coordinate adequate health promotion campaigns to educate, inform, and enhance the trust in institutions, to safeguard the welfare of the public during future epidemics and disasters.

## Figures and Tables

**Table 1:** Participant characteristics by different levels of knowledge and attitude, a national wide cross-sectional survey in China, 2020 (N=1426)

Variable	N (%)	Knowledge N (%)			Attitude N (%)		
		poor	moderate	good	negative	neutral	positive
Total	1426	66 (4.6)	630 (44.2)	730 (51.2)	103 (7.2)	667 (46.8)	656 (46.0)
**Gender**							
Male	869 (60.9)	42 (4.8)	406 (46.7)	421 (48.4) *	59 (6.7)	415 (47.8)	395(45.5)
Female	557 (39.1)	24 (4.3)	224 (40.2)	309 (55.5)	44 (7.9)	252 (45.2)	261 (46.9)
**Age (years)**							
16~25	911 (63.9)	32 (3.5)	399 (43.8)	480 (52.7) **	63 (6.9)	439 (48.2)	409 (44.9)
26~35	367 (25.7)	16 (4.4)	178 (48.5)	173 (47.1)	28 (7.6)	172 (46.9)	167 (45.5)
36~45	133 (9.3)	18 (13.5)	48 (36.1)	67 (50.4)	11 (8.3)	53 (39.8)	69 (51.9)
>45	15 (1.1)	0 (0.0)	5 (33.3)	10 (66.7)	1 (6.7)	3 (20.0)	11 (73.3)
**Legal marital status**							
Never married	1236 (86.7)	53 (4.3)	552 (44.7)	631 (51.1)	83 (6.7)	587 (47.5)	566 (45.8)
Ever married/engaged	190 (13.3)	13 (6.8)	78 (41.1)	99 (52.1)	20 (10.5)	80 (42.1)	90 (47.4)
**Highest educational attainment**							
High school or below	436 (30.6)	21 (4.8)	192 (44.0)	223 (51.2)	28 (6.4)	219 (50.2)	189 (43.4)
Some college	163 (11.4)	1 (0.6)	66 (40.5)	96 (58.9)	11 (6.7)	79 (48.5)	73 (44.8)
Bachelor's or higher	827 (58.0)	44 (5.3)	372 (45.0)	411 (49.7)	64 (7.7)	369 (44.6)	394 (47.7)
**Annual income (USD)**							
< $2000	894 (62.7)	33 (3.7)	409 (45.7)	452 (50.6)	59 (6.6)	434 (48.5)	401 (44.9)
$2000- $5000	255 (17.9)	16 (6.3)	110 (43.1)	129 (50.6)	15 (5.9)	116 (45.5)	124 (48.6)
$5000-$10000	128 (9.0)	8 (6.3)	49 (38.2)	71 (55.5)	14 (10.9)	58 (45.3)	56 (43.8)
> $10000	149 (10.4)	9 (6.0)	62 (41.6)	78 (52.3)	15 (10.1)	59 (39.6)	75 (50.3)
**Original country**							
Asia	332 (23.3)	31 (9.3)	150 (45.2)	151 (45.5) **	19 (5.7)	141 (42.5)	172 (51.8)
Europe	20 (1.4)	0 (0.0)	13 (65.0)	7 (35.0)	2 (10.0)	5 (25.0)	13 (65.0)
South America	1 (0.1)	0 (0.0)	0 (0.0)	1 (100.0)	0 (0.0)	1 (100.0)	0 (0.0)
North America	18 (1.3)	0 (0.0)	5 (27.8)	13 (72.2)	0 (0.0)	6 (33.3)	12 (66.7)
Oceania	5 (0.4)	0 (0.0)	3 (60.0)	2 (40.0)	0 (0.0)	2 (40.0)	3 (60.0)
Africa	1050 (73.6)	35 (3.3)	459 (43.7)	556 (53.0)	82 (7.8)	512 (48.8)	456 (43.4)
**Religion**							
Christianity	918 (64.4)	21 (2.3)	405 (44.1)	492 (53.6) **	65 (7.0)	454 (49.5)	399 (43.5)
Islam	325 (22.8)	35 (10.8)	147 (45.2)	143 (44.0)	24 (7.4)	133 (40.9)	168 (51.7)
Buddhism	17 (1.2)	1 (5.9)	11 (64.7)	5 (29.4)	2 (11.8)	10 (58.8)	5 (29.4)
Other	53 (3.7)	4 (7.6)	19 (35.8)	30 (56.6)	3 (5.6)	25 (47.2)	25 (47.2)
None	113 (7.9)	5 (4.4)	48 (42.5)	60 (53.1)	9 (8.0)	45 (39.8)	59 (52.2)
**Reasons for migration**							
Business	373 (26.2)	38 (10.2)	173 (46.4)	162 (43.4) **	34 (9.1)	146 (39.1)	193 (51.8) *
Study	872 (61.2)	25 (2.9)	379 (43.5)	468 (53.7)	58 (6.7)	446 (51.1)	368 (42.2)
Employment	117 (8.2)	1 (0.9)	46 (39.3)	70 (59.8)	8 (6.8)	47 (40.2)	62 (53.0)
Tourism	49 (3.4)	1 (2.0)	25 (51.0)	23 (46.9)	3 (6.1)	20 (40.8)	26 (53.1)
Visiting relatives	15 (1.1)	1 (6.7)	7 (46.7)	7 (46.7)	0 (0.0)	8 (53.3)	7 (46.7)
**Cumulative stay in China**							
1-6 months	187 (13.1)	10 (5.3)	85 (45.5)	92 (49.2)	12 (6.4)	82 (43.9)	93 (49.7)
7-12 months	135 (9.5)	8 (5.9)	71 (52.6)	56 (41.5)	10 (7.4)	63 (46.7)	62 (45.9)
One year and above	1104 (77.4)	48 (4.3)	474 (42.9)	582 (52.7)	81 (7.3)	522 (47.3)	501 (45.4)
**Stay in China between December 2019 and February 2020**							
1 day- 2 weeks	23 (1.6)	4 (17.4)	10 (43.5)	9 (39.1) **	2 (8.7)	10 (43.5)	11 (47.8)
2 weeks- 1 month	25 (1.8)	4 (16.0)	11 (44.0)	10 (40.0)	2 (8.0)	14 (56.0)	9 (36.0)
1 month- 2 months	242 (17.0)	23 (9.5)	98 (40.5)	121 (50.0)	26 (10.7)	108 (44.6)	108 (44.6)
3 months	1136 (79.7)	35 (3.1)	511 (45.0)	590 (51.9)	73 (6.4)	535 (47.1)	528 (46.5)
**Living arrangement in China**							
Hotel	82 (5.8)	8 (9.8)	52 (63.4)	22 (26.8) **	8 (9.7)	29 (35.4)	45 (54.9) *
Guest apartment	34 (2.4)	4 (11.8)	11 (32.4)	19 (55.9)	0 (0.0)	16 (47.1)	18 (52.9)
Purchased apartment	14 (1.0)	3 (21.4)	4 (28.6)	7 (50.0)	3 (21.4)	7 (50.0)	4 (28.6)
Rental apartment	503 (35.3)	24 (4.8)	208 (41.4)	271 (53.9)	37 (7.4)	210 (41.7)	256 (50.9)
Staff/student dormitory	76 (53.9)	23 (3.0)	343 (44.7)	402 (52.3)	53 (6.9)	390 (50.8)	325 (42.3)
No fixed residence	25 (1.8)	4 (16.0)	12 (48.0)	9 (36.0)	2 (8.0)	15 (60.0)	8 (32.0)
**Health insurance in China**							
Yes	1190 (83.5)	54 (4.5)	522 (43.9)	614 (51.6)	86 (7.2)	570 (47.9)	534 (44.9)
No	236 (16.6)	12 (5.1)	108 (45.8)	116 (49.1)	17 (7.2)	97 (41.1)	122 (51.7)
**Having had the following diseases in the past year**							
Flu	335 (23.5)	12 (3.6)	134 (40.0)	189 (56.4) **	31 (9.2)	154 (46.0)	150 (44.8)
Tuberculosis	7 (0.5)	3 (42.8)	3 (42.8)	1 (14.3)	1 (14.3)	2 (28.6)	4 (57.1)
Typhoid fever	8 (0.6)	3 (37.5)	1 (12.5)	4 (50.0)	0 (0.0)	2 (25.0)	6 (75.0)
Infectious diarrhea	9 (0.6)	2 (22.2)	4 (44.4)	3 (33.3)	2 (22.2)	3 (33.3)	4 (44.5)
HIV/STI	8 (0.5)	2 (25.0)	5 (62.5)	1 (12.5)	1 (12.5)	2 (25.0)	5 (62.5)
None	1069 (75.0)	47 (4.4)	486 (45.5)	536 (50.1)	71 (6.6)	500 (46.8)	498 (46.6)
**Being diagnosed as the COVID-19**							
Yes	13 (0.9)	4 (30.8)	5 (38.5)	4 (30.8) **	3 (23.0)	5 (38.5)	5 (38.5)
No	1413 (99.1)	62 (4.4)	625 (44.2)	726 (51.4)	100 (7.0)	662 (46.9)	651 (46.1)

Note:* Chi-square test, *P<0.05, **P<0.001.

**Table 2:** Knowledge, attitude and experiences related to the COVID-19 outbreak among international migrants, a national wide cross-sectional survey in China, 2020 (N=1426)

Variable	N	%
**Knowledge**		
***The symptoms after contracting COVID-19***		
Correct	1055	74.0
Incorrect	371	26.0
***The signs indicating seeking health care immediately***		
Correct	1217	85.3
Incorrect	209	14.7
***The outcomes caused by COVID-19***		
Correct	1256	88.1
Incorrect	170	11.9
***Transmission routes***		
Correct	1370	96.1
Incorrect	56	3.9
***Quarantine period***		
Correct	1340	94.0
Incorrect	86	6.0
***Availability of specific drug or vaccine***	760^a^	53.3
No drug	1020	71.5
No vaccine	1124	78.8
***Prevention strategies on COVID-19***	729^b^	51.1
Wash hands frequently	1319	92.5
Do not go to crowded places	1240	87.0
Wear mask	1310	91.9
Reduced contact to wild animals	923	64.7
Keep room well ventilated	939	65.9
Stay indoors and avoid going out	1173	82.3
**Attitude**		
***Being confident of knowing how to protect yourself from the COVID-19***		
Strongly disagree	16	1.1
Disagree	138	9.7
Agree	392	27.5
Strongly agree	880	61.7
***Worry about contracting COVID-19***		
Strongly disagree	628	44.0
Disagree	205	14.4
Agree	291	20.4
Strongly agree	302	21.2
***Worry about loved ones/friends contracting COVID-19***		
Strongly disagree	435	30.5
Disagree	166	11.6
Agree	269	18.9
Strongly agree	556	39.0
***Feel helpless to prevent COVID-19***		
Strongly disagree	356	25.0
Disagree	566	39.7
Agree	390	27.4
Strongly agree	114	8.0
***Being confident the COVID-19 will end soon***		
Strongly disagree	59	4.1
Disagree	108	7.6
Agree	701	49.2
Strongly agree	558	39.1
**Experiences**		
***Might have been**** infected with COVID-19*		
Yes	39	2.7
No	1300	91.2
Unsure	87	6.1
***Plan to seek medical screening for diagnosis***		
Yes	44	34.9
No	46	36.5
Unsure	36	28.6
***Reasons for not testing or seeking diagnostic treatment***		
Fear of the virus	55	43.7
Avoid quarantine	40	31.8
The virus is not serious	16	12.7
My symptoms are not that serious	43	34.1
Don't know where to get the service	16	12.7
Unable to afford money for transport to clinic	13	10.3
Treatment is meaningless	4	3.2
Difficult to make an appointment with a doctor	16	12.7
Afraid that people will look down on me	17	13.5
***Plan to leave China because of COVID-19***		
Yes	152	10.7
No	962	67.5
Unsure	312	21.9

^a^ refers to the number (%) of individuals who answered correct for both of the questions; ^b^ refers to the number (%) of individuals who answered correct for all of the questions.

**Table 3:** Trust in Chinese institutions and groups among international migrants, a national wide cross-sectional survey in China, 2020 (N=868)

Variables	N (%)	Knowledge N (%)			Attitude N (%)		
		poor	moderate	good	negative	neutral	positive
**General rating**							
High trust	455 (52.4)	7 (1.5)	216 (47.5)	232 (51.0) **	16 (3.5)	241 (53.0)	198 (43.5) **
Moderate trust	253 (29.2)	8 (3.2)	95 (37.5)	150 (59.3)	21 (8.3)	127 (50.2)	105 (41.5)
Low trust	160 (18.4)	13 (8.1)	65 (40.6)	82 (51.3)	25 (15.6)	77 (48.1)	58 (36.3)
**The Central Government**							
High trust	540 (62.2)	11 (2.0)	242 (44.8)	287 (53.2)	23 (4.2)	285 (52.8)	232 (43.0) *
Moderate trust	168 (19.4)	7 (4.2)	67 (39.8)	94 (56.0)	20 (11.9)	88 (52.4)	60 (35.7)
Low trust	160 (18.4)	10 (6.2)	67 (41.9)	83 (51.9)	19 (11.9)	72 (45.0)	69 (43.1)
**The department that handles health**							
High trust	560 (64.5)	9 (1.6)	251 (44.8)	300 (53.6) *	22 (3.9)	296 (52.9)	242 (43.2) **
Moderate trust	176 (20.3)	13 (7.4)	69 (39.2)	94 (53.4)	20 (11.4)	87 (49.4)	69 (39.2)
Low trust	132 (15.2)	6 (4.5)	56 (42.5)	70 (53.0)	20 (15.1)	62 (47.0)	50 (37.9)
**The department that handles immigration**							
High trust	592 (68.2)	10 (1.7)	263 (44.4)	319 (53.9) *	32 (5.4)	304 (51.4)	256 (43.2)
Moderate trust	154 (17.7)	9 (5.8)	61 (39.6)	84 (54.6)	16 (10.4)	81 (52.6)	57 (37.0)
Low trust	122 (14.1)	9 (7.4)	52 (42.6)	61 (50.0)	14 (11.5)	60 (49.2)	48 (39.3)
**The hospital system**							
High trust	578 (66.6)	7 (1.2)	261 (45.2)	310 (53.6) **	27 (4.7)	300 (51.9)	251 (43.4) *
Moderate trust	159 (18.3)	14 (8.8)	59 (37.1)	86 (54.1)	18 (11.3)	76 (47.8)	65 (40.9)
Low trust	131 (15.1)	7 (5.3)	56 (42.7)	68 (52.0)	17 (13.0)	69 (52.6)	45 (34.4)
**Doctors and medical professionals**							
High trust	605 (69.7)	9 (1.5)	271 (44.8)	325 (53.7) **	26 (4.3)	315 (52.1)	264 (43.6) **
Moderate trust	150 (17.3)	12 (8.0)	59 (39.3)	79 (52.7)	19 (12.7)	72 (48.0)	59 (39.3)
Low trust	113 (13.0)	7 (6.2)	46 (40.7)	60 (53.1)	17 (15.0)	58 (51.3)	38 (33.6)
**The information you are receiving about the COVID-19**							
High trust	450 (51.9)	9 (2.0)	210 (46.7)	231 (51.3)	16 (3.5)	242 (53.8)	192 (42.7) **
Moderate trust	200 (23.0)	10 (5.0)	84 (42.0)	106 (53.0)	19(9.5)	94 (47.0)	87 (43.5)
Low trust	218 (25.1)	9 (4.1)	82 (37.6)	127 (58.3)	27 (12.4)	109 (50.0)	82 (37.6)
**The police**							
High trust	539 (62.1)	8 (1.5)	239 (44.3)	292 (54.2) *	24 (4.4)	278 (51.6)	237 (44.0) *
Moderate trust	163 (18.8)	8 (4.9)	67 (41.1)	88 (54.0)	18 (11.0)	86 (52.8)	59 (36.2)
Low trust	166 (19.1)	12 (7.2)	70 (42.2)	84 (50.6)	20 (12.0)	81 (48.8)	65 (39.2)
**The Chinese people**							
High trust	424 (48.9)	8 (1.9)	204 (48.1)	212 (50.0) *	19 (4.5)	214 (50.5)	191 (45.0) *
Moderate trust	184 (21.2)	7 (3.8)	70 (38.0)	107 (58.2)	15 (8.1)	96 (52.2)	73 (39.7)
Low trust	260 (30.0)	13 (5.0)	102 (39.2)	145 (55.8)	28 (10.8)	135 (51.9)	97 (37.3)

Note: *Chi-square test, *P<0.05, **P<0.001.

**Table 4:** Information channels and preferences related to COVID-19 among international migrants, a national wide cross-sectional survey in China, 2020 (N=1426)

Variables	N	%
**Channels for receiving information**		
Wechat	1348	94.5
Microblog	207	14.5
Website	687	48.2
Television	474	33.2
APP related to news	435	30.5
Community	463	32.5
Friends	849	59.5
Newspaper	233	16.3
Leaflet	62	4.4
Others	50	3.5
**Preferred channels for receiving information**		
Wechat	1335	93.6
Microblog	205	14.4
Website	627	44.0
Television	454	31.8
APP related to news	412	28.9
Community	348	24.4
Friends	492	34.5
Newspaper	288	20.2
Leaflet	100	7.0
Others	29	2.0
**Preferred information to know**		
Where the virus came from	927	65.0
How the disease is transmitted	676	47.4
How to prevent the disease	834	58.5
How to cure the disease	952	66.8
The symptoms of the disease	636	44.6
Status and trend of epidemic	759	53.2
Psychological support or counseling	535	37.5
**Barriers to receive medical services**		
Language communication	786	55.1
Waiting time is too long	191	13.4
Not familiar with the medical procedure	267	18.7
Do not understand the drug instruction	144	10.1
Discriminated/insulted by doctor/nurse	60	4.2
Suffer to discrimination/insult of other patients	60	4.2
Can't receive medical services due to foreign identity	75	5.3
Problems related with medical insurance reimbursement	112	7.9
Do not have the above difficulties	483	33.9
**The quality of medical service regarding COVID-19 in China**		
Poor	301	21.1
Moderate	313	22.0
Good	812	56.9

**Table 5:** Factors correlated with knowledge and attitude among international migrants, a national wide cross-sectional survey in China, 2020 (N=1426)

Characteristics	Knowledge		Attitude	
	cOR (95%CI)	aOR (95%CI) ^#^	cOR (95%CI)	aOR (95%CI) ^#^
Health insurance in China (ref no.)	1.1 (0.8-1.5)	1.0 (0.8-1.3)	1.0 (0.8-1.3)	1.1 (0.8-1.4)
Having had infection diseases in the past year (ref no.)	1.1 (0.9-1.5)	1.0 (0.8-1.3)	0.8 (0.7-1.0)	0.9 (0.7-1.1)
Receiving information through social media (Wechat/Microblog) (ref no.)	2.0 (1.2-3.2) *	2.0 (1.2-3.2) *	1.3 (0.8-2.1)	1.4 (0.9-2.2)
Receiving information through Internet (website/app related to news) (ref no.)	1.5 (1.2-1.8) **	1.4 (1.2-1.8) **	1.0 (0.8-1.3)	1.0 (0.9-1.3)
Receiving information through television (television/newspaper) (ref no.)	1.1 (0.9-1.4)	1.1 (0.9-1.4)	1.1 (0.9-1.3)	1.1 (0.9-1.3)
Receiving information through community (community/friends/leaflet) (ref no.)	1.4 (1.2-1.8) **	1.5 (1.2-1.8) **	0.9 (0.8-1.2)	1.0 (0.8-1.2)
Encountering language barriers when receiving medical services ^a^ (ref no.)	0.8 (0.7-1.0) *	0.8 (0.7-1.0) *	0.9 (0.7-1.1)	0.9 (0.7-1.0)
Encountering medical system barriers when receiving medical services ^b^ (ref no.)	0.9 (0.7-1.2)	0.9 (0.7-1.2)	0.9 (0.7-1.1)	0.8 (0.7-1.1)
Encountering discrimination barriers when receiving medical services^ c^ (ref no.)	1.0 (0.7-1.5)	1.0 (0.7-1.5)	0.8 (0.5-1.1)	0.8 (0.5-1.1)
**The quality of medical service regarding COVID-19 in China**				
good	0.8 (0.6-1.1)	0.8 (0.6-1.1)	1.7 (1.3-2.2) **	1.7 (1.3-2.2) **
moderate	0.9 (0.7-1.4)	1.0 (0.7-1.4)	1.4 (1.1-1.9) *	1.5 (1.1-2.0) *
poor	*ref*	*ref*	*ref*	*ref*
**The Central Government**				
High trust	1.1 (0.8-1.6)	1.1 (0.8-1.6)	1.5 (1.1-2.2) *	1.6 (1.1-2.2) *
Moderate trust	1.2 (0.8-1.9)	1.2 (0.8-1.8)	0.6 (0.4-1.0) *	0.7 (0.4-1.0)
Low trust	*ref*	*ref*	*ref*	*ref*
**The department that handles health**				
High trust	1.1 (0.7-1.6)	1.1 (0.7-1.6)	1.6 (1.1-2.3) *	1.6 (1.1-2.3) *
Moderate trust	1.0 (0.6-1.5)	0.9 (0.6-1.5)	0.8 (0.5-1.3)	0.8 (0.5-1.3)
Low trust	*ref*	*ref*	*ref*	*ref*
**The department that handles immigration**				
High trust	1.3 (0.9-1.9)	1.3 (0.9-1.9)	1.5 (1.0-2.2) *	1.5 (1.0-2.2) *
Moderate trust	1.2 (0.8-2.0)	1.2 (0.8-2.0)	0.9 (0.6-1.5)	1.0 (0.6-1.5)
Low trust	*ref*	*ref*	*ref*	*ref*
**The hospital system**				
High trust	1.2 (0.8-1.7)	1.1 (0.8-1.6)	1.7 (1.2-2.5) *	1.8 (1.2-2.6) *
Moderate trust	1.0 (0.6-1.6)	1.0 (0.6-1.6)	1.1 (0.7-1.7)	1.1 (0.7-1.8)
Low trust	*ref*	*Ref*	*ref*	*ref*
**Doctors and medical professionals**				
High trust	1.1 (0.8-1.7)	1.0 (0.7-1.5)	2.0 (1.4-3.0) **	2.1 (1.4-3.1) **
Moderate trust	0.9 (0.6-1.5)	0.9 (0.5-1.4)	1.2 (0.7-1.9)	1.2 (0.7-1.9)
Low trust	*ref*	*ref*	*ref*	*ref*
**The information you are receiving about the COVID-19**				
High trust	0.8 (0.6-1.1)	0.8 (0.6-1.1)	1.9 (1.4-2.5) **	1.8 (1.3-2.5) **
Moderate trust	0.8 (0.5-1.2)	0.8 (0.6-1.2)	1.2 (0.9-1.8)	1.2 (0.9-1.8)
Low trust	*ref*	*ref*	*ref*	*ref*
**The police**				
High trust	1.3 (0.9-1.8)	1.2 (0.9-1.8)	1.7 (1.2-2.3) *	1.7 (1.2-2.4) *
Moderate trust	1.2 (0.8-1.8)	1.2 (0.8-1.8)	0.8 (0.5-1.2)	0.8 (0.5-1.2)
Low trust	*ref*	*ref*	*ref*	*ref*
**The Chinese people**				
High trust	1.1 (0.9-1.4)	0.8 (0.6-1.1)	1.7 (1.3-2.3) **	1.7 (1.3-2.3) **
Moderate trust	1.4 (1.0-1.9) *	1.1 (0.8-1.6)	0.8 (0.6-1.2)	0.8 (0.6-1.2)
Low trust	*ref*	*ref*	*ref*	*ref*

Note: * *P*<0.05, **P<0.001; The outcome of knowledge was categorized into poor, moderate and good; The outcome of attitude was categorized into negative, neutral and positive; #Multivariable ordinal logistic regression adjusted for gender, age, legal marital status, highest educational attainment, annual income (USD), original country, religion, reasons for migration; OR: crude odds ratio; aOR: adjusted odds ratio; CI: confidence interval; ^a^ Language barriers include: language communication barriers, and do not understand the drug instruction; ^b^ Medical system barriers include: too long waiting time, not familiar with the medical procedure of seeking care, cannot receive medical services due to foreign identity, having problems related with medical insurance reimbursement; ^c^ Discrimination barriers include: discriminated/insulted by doctor/nurse and suffer to discrimination/insult of other patients.

## Abbreviations

COVID-19: novel coronavirus disease
OR: crude odds ratio
CI: confidence interval

## Supplementary material

Knowledge and attitude scales related to COVID-19.
